# Changes in the bulk soil after fresh corn grown with organic and inorganic fertilizer application

**DOI:** 10.1371/journal.pone.0326730

**Published:** 2025-07-10

**Authors:** Riri Dayang Sari Risman, Kiriya Sungthongwises, Supanath Kanjanawattanawong

**Affiliations:** 1 Agronomy Section, Faculty of Agriculture, Khon Kaen University, Khon Kaen, Thailand; 2 Horticulture Section, Faculty of Agriculture, Khon Kaen University, Khon Kaen, Thailand; Graphic Era Institute of Technology: Graphic Era Deemed to be University, INDIA

## Abstract

The effects of different fertilizer applications on crop growth, soil health, and microbial communities are critical for sustainable agriculture. Positive interactions between crop roots and their associated microbiomes are essential to improve nutrient availability and promote plant growth. Therefore, this study aimed to investigate the changes in bulk soil chemical properties and diversity of phosphate-solubilizing microorganisms after growing three fresh corn plants under the application of vermicompost, black soldier flies, and inorganic fertilizers. Fresh corn yield and soil samples were collected from purple waxy, pink waxy, and sweet corn grown under field conditions. The capacity to solubilize mineral phosphate and indole acetic acid was also determined using a spectrophotometer. The results showed that organic and inorganic fertilizers can maintain the ear-fresh weight of the three fresh corn varieties and tend to increase some soil chemicals after growth. Application of inorganic fertilizer and black soldier flies mixed with inorganic fertilizer resulted in the highest ear fresh weight, with 6,291.30 and 5,887.40 kg ha^-1^, respectively. Moreover, the soil pH, available phosphorus, and copper tended to increase, whereas zinc and chromium decreased. However, fertilizer management did not affect the diversity of the phosphate-solubilizing microorganisms. In addition, the three phosphate-solubilizing fungal isolates were similar to the type strain of Candida tropicalis. The phosphate-solubilizing fungi isolate potentials were not significantly different in AlPO_4_, and FePO_4_ solubilizing. Only two PSF isolates from purple waxy produced IAA hormone between 462.81–562.81 mg l^-1^.

## Introduction

Fresh corn (*Zea mays* L.) is a widely cultivated crop of significant economic and nutritional importance. Nutrient availability is a critical factor in the growth and yield of crops, particularly in sustainable agricultural systems. Among the various essential nutrients, nitrogen (N) and phosphorus (P) play important roles in vegetative, and reproductive growth, respectively. Phosphorus enhances root, energy transfer, and overall metabolic processes at the beginning of plant growth [1]. However, the bioavailability of P in soil is often limited because of its tendency to form insoluble complexes, rendering it inaccessible to plants [2]. Fertilizer application affects plant growth, soil fertility, and microbial community composition [3]. The soil microbial community plays a crucial role in plant growth, yield components, and yield, compound decomposition, and other transformations [4]. In practice, greater microbial diversity enhances soil fertility and helps maintain nutrient balance, ultimately supporting improved corn growth and yield. Fertilizer application alters plant dependence on increasing resources by increasing soil nutrient levels [5]. Excessive use of inorganic fertilizers over the long term is unsustainable and can lead to poor soil health and nutrient balance. Repeated application of fertilizers can cause certain microorganisms to proliferate, leading to a reduction in microbial diversity, while other beneficial microbes may be suppressed [6]. This imbalance can negatively affect soil fertility, reduce nutrient cycling efficiency, and ultimately harm plant growth and ecosystem health. Sustainable practices, including the use of organic fertilizers, can increase phosphate-solubilizing microorganisms (PSM) in the soil, which can help restore balance and maintain soil health over time [7].

Phosphate-solubilizing microorganisms are crucial for supplementing P and plant growth-promoting hormones [8]. Auxin, cytokinin, and gibberellin affect plant germination, root and shoot development, xylem differentiation, and flowering [9]. Organic acids, phosphatase enzymes, and complexing agents produced by plants and microorganisms in soil can solubilize insoluble P compounds [10]. However, microorganisms are involved in pH regulation by acid production, ion chelation, and exchange reactions [11]. Bacteria and fungi are considered to be the most important factors that help plant nutrient acquisition and improve plant health. Many species of microorganisms have been identified, particularly in the soil rhizosphere. Phosphate-solubilizing bacteria (PSB) showed 1−50% of the total microbial population, while phosphate-solubilizing fungi (PSF) account for 0.1–0.5% [12]. Generally, bacterial species are more efficient at P solubilization than fungi [13]. Fungi are crucial in the breakdown and utilization of both easily accessible and more complex carbon sources derived from litter, a process in which they play a more active role than bacteria, contributing significantly to soil formation [14]. Moreover, fungi have a high capacity to adapt to various adverse abiotic conditions such as salinity, drought, heavy metals, and extreme pH [15].

However, the excessive use of inorganic fertilizers can lead to soil degradation, reduced microbial diversity, and nutrient imbalances, making it unsustainable in the long term [16]. Integrated nutrient management strategies that combine organic and inorganic fertilizers have been found to improve soil properties and enhance microbial activity, particularly in acidic soils where P availability is naturally low [17]. The incorporation of vermicompost and black soldier fly (BSF) frass as organic amendments has been reported to improve soil organic matter content, increase microbial diversity, and support P solubilization [18,19]. The selection of sweet, purple waxy, and pink waxy corn in this study was based on their commercial suggestion [20,21], agronomic potential, and nutritional value. Previous studies have shown that these corn types exhibit varied responses to soil conditions and nutrient availability [22]. Understanding how different organic fertilizer strategies, including inorganic fertilizers, affect soil properties and the activity of PSMs in corn cultivation systems is crucial for optimizing crop production and promoting sustainable agricultural practices. This study aimed to evaluate the changes in bulk soil properties and diversity of phosphate-solubilizing microorganisms after growing fresh corn the short-term application of vermicompost and BSF mixed with inorganic fertilizers. To investigate the interactions between soil amendments and microbial communities. This study was initiated to provide insights into the potential benefits of integrating organic and inorganic fertilizers in corn production systems.

## Materials and methods

### Experimental site and yield determination

The field trials were established in the Chonnabot district, Khon Kaen province, Northeast Thailand (latitude 16.4779°N and longitude 102.8558°N, 166.43 m above sea level). The land has acidic sandy soil with a total rainfall during the crop growth period of 2023−2024 850 mm and 868 mm. The maximum (32 °C and 30 °C) and minimum (25 °C and 24 °C) temperatures are recorded in 2023 and 2024, respectively. The study was conducted on private farmer fields, no specific permits were required for field access. The soil was amended with cattle manure at a rate of 6,250 kg ha^-1^ (recommended by the Department of Agriculture, Thailand) and left to incubate for two weeks before planting the three varieties of fresh corn (pink waxy, purple waxy, and sweet corn).

The plants were spaced 65 cm apart between rows and 25 cm within rows, with planting depth of 3−4 cm. Each plot measured 2 × 10 m, with 12 plots per variety across two crop cycles. Irrigation was provided to maintain field capacity in case of intermittent drought affecting the fresh corn. Four fertilizer treatments were adapted with inorganic fertilizer suggestion of the Department of Agriculture, Thailand for fresh corn production, as follows: T1) no fertilizer (control), T2) 6,250 kg ha^-1^ of vermicompost combined with 95.31 kg N ha^-1^, 23.44 kg P ha^-1^, and 23.44 kg K ha^-1^, T3) 6,250 kg ha^-1^ of black soldier fly (BSF) with 95.31 kg N ha^-1^, 23.44 kg P ha^-1^, and 23.44 kg K ha^-1^, and T4) 190.63 kg N ha^-1^, 46.88 kg P ha^-1^, and 46.88 kg K ha^-1^. The experiment utilized a strip-plot design with three replications. Fertilizers were applied on two occasions during the study. The initial application occurred 20 days after germination (DAG), and the next application was between 40 and 45 DAG. Weeding was carried out manually using a hoe, four weeks after planting and as needed thereafter. Fresh corn yield was determined by manual harvesting from the two central rows of each plot. Several parameters, including ear length, ear diameter, ear fresh weight, 1000 seed weight, and harvest index (HI), were recorded at the harvest stage.

### Soil sample collection

Before and after harvesting, soil samples were taken from the experimental site for chemical analysis. Within each site, soil samples from the surface 0–30 cm were collected from ten points a soil auger and mixed as one composite sample for soil chemical analysis. Air-dried soil samples were crushed to measure soil nutrients and passed through a 0.25 mm sieve. Soil pH was measured using a pH meter [23], organic matter (OM) by the Walkley and Black method [24], electrical conductivity (EC) using an EC meter [25], total N by the Kjeldahl method [26], available P by Bray II and the molybdenum-blue method [27], exchangeable potassium (K), calcium (Ca), magnesium (Mg) by 1 N NH_4_OAc, pH 7, and flame photometry method, iron (Fe), manganese (Mn), Cu, and Zn by diethylenetriaminepentaacetic acid (DTPA) pH 7.3, atomic absorption spectrophotometry, and total Cr, arsenic (As), cadmium (Cd), and lead (Pb) by wet digestion and inductively coupled plasma optical emission spectroscopy (ICP-OES). Soil samples for PSMs isolation were collected from bulk soil at 0–10 cm, with 10 points plot^-1^ (n = 10) in sterilized plastic bags and preserved in a cool box at 4°C after the fresh corn was harvested.

### Isolation of PSMs isolates by enrichment culture

Phosphate-solubilizing microorganisms were isolated from soil samples using the National Botanical Research Institute’s Phosphate (NBRIP) growth media [28]. One liter of NBRIP include 10 g glucose, 0.2 g KCl, 5 g MgCl_2_·6H_2_O, 0.25 g MgSO_4_·7H_2_O and 0.1 g (NH_4_)_2_SO_4_. Insoluble P, AlPO_4_, FePO_4_, and Ca_3_(PO_4_)_2_ were modified with NBRIP medium, to measure phosphate solubilization. The pH of insoluble P was adjusted to 7.0 and autoclaved separately with sugar and stock solutions. After autoclaving, the sterile ingredients were mixed in laminar airflow. Erlenmeyer flasks containing 50 ml of the medium with soil samples were incubated for one week at 30 °C in an incubator shaker at 150 cycles min^-1^. Every week, 5 ml of inoculant was transferred into 50 ml Erlenmeyer flasks with a new liquid medium for 6 weeks. At the end of six weeks in NBRIP growth liquid medium, aliquots of each dilution were spread over the NBRIP solid medium. Phosphate-solubilizing microorganism colonies were selected from the plates based on the appearance of a clear halo, and clones were further purified for mineral phosphate solubilization and IAA production.

### Mineral phosphate solubilization

Phosphate-solubilizing microorganism isolates were tested for their ability to solubilize phosphate by determining the solubilizing activity of each isolate in four replicates using the molybdenum-blue method [29]. The isolates were grown in NBRIP liquid media containing different insoluble forms of phosphate (AlPO_4_, Ca_3_(PO_4_)_2_, and FePO_4_) for three days at 30 °C in an incubator shaker at a medium speed (150 cycles min^-1^). Solubilization efficiencies were determined by reactions with ammonium molybdate for phosphorus compounds, such as ammonium phosphomolybdate, and reduced to molybdenum blue with ascorbic acid. The isolates were incubated for 30 min at room temperature for color development. The light absorption was measured at a wavelength of 880 nm using a Shimadzu UV-120–01 spectrophotometer. The concentration of phosphate solubilization activity was compared to a standard curve of KH_2_PO_4_ at concentrations ranging from 0 to 0.9 ml l^-1^.

### Analysis of indole acetic acid production (IAA)

Phosphate-solubilizing microorganism isolates were selected for IAA production analysis [30]. Five µl of selective PSMs isolates were tested with 1 ml of Tris-TMRT reagent comprising 0.2 g L^-1^ CaCl_2_·2H_2_O, 10 g L^-1^ D-mannitol, 0.2 g L^-1^ MgSO_4_·7H_2_O, 0.2 g L^-1^ yeast extract, 0.061 g L^-1^ L-Tryptophan and 1.21 g L^-1^ Tris-base in the dark condition at 25 °C for ten days. Each selective PSM isolated with Tris-TMRT reagent was synthesized with 1 ml of 0.01 M FeCl_3_ in 35% HClO_4_ for 30 min in the dark at 25 °C. The red color of the isolated mixtures indicates that the isolate positively produced IAA. To determine the volume of IAA production, the positively isolated strain with red color was cultivated in 50 ml of Luria-Bertani medium (LB), consisting of 5 g L^-1^ NaCl, 10 g L^-1^ Tryptone, and 5 g L^-1^ yeast extract at 30 °C for three days. The solution of selective PSM isolates was centrifuged at 14,000 rpm and the supernatant was suspended. The supernatant was transferred and synthesized with 1 ml of 0.01 M FeCl_3_ in 35% HClO_4_ for 30 min in the dark at 25 °C. Light absorption was measured at 530 nm using a Shimadzu UV-120–01 spectrophotometer. Then, the concentration of indole acetic acid produced by the PSM was compared to a standard curve of IAA at concentrations ranging from 0 to 150 µg ml^-1^.

### Isolation of DNA for polymerase chain reaction (PCR)

Isolation of PSM isolated DNA for PCR and Identification of PSF were conducted at a commercial laboratory, Thailand Bioresource Research Center (TBRC), in Bangkok, Thailand. DNA isolation was carried out by boiling the cells with lysis buffer according to the methods described by Manitis et al. [31], with slight modifications. A loopful of yeast cells was transferred to a 1.5 ml Eppendorf tube. Lysis buffer (100 µL) was then added. The cell suspensions were boiled in a water bath or metal block bath for 15 min. Next, 100 µl of 2.5 M potassium acetate (pH 7.5) was added, placed on ice for 1 h, and centrifuged at 14,500 rpm for 5 min. The supernatant was extracted twice with 100 µL chloroform: isoamyl alcohol (24:1 v/v). DNA was precipitated with isopropanol, incubated at 20 °C for 10 min, and centrifuged at 14,500 rpm for 15 min. The DNA pellet was rinsed with 70% and 90% ethanol, and then dried for up (15–30 min at room temperature). Dried DNA was dissolved in 30 µL nanopore water (S1 File).

### Identification of phosphate solubilizing fungi (PSF)

The divergent D1-D2 domain of *26S* rDNA was amplified with primers NL-1 (5’- GCA TAT CAA TAA GCG GAG GAA AAG-3’) and NL4 (5’-GGT CCG TGT TTC AAG ACG G-3’) [32]. The specific make and model of the PCR thermal cycler is Biorad model T100. Amplification was carried out in a 100 µL reaction mixture containing 100 ng of genomic DNA, 5 U µl^-1^ of Taq polymerase, 2.5 mM of each dNTP, 10 pM of each primer, 10X Taq buffer, and 25 mM MgCl2. The reaction was pre-denatured at 94 °C for 5 min, followed by 30 PCR cycles of denaturation at 94 °C for 1 min, annealing at 52 °C for 1.30 min and extension at 72 °C for 2.30 min, followed by a final extension at 72 °C for 10 min. The PCR product was analyzed by agarose gel electrophoresis and purified using the GenepHlowTM Gek/PCR Kit (Geneaid Biotech Ltd., Taiwan). The amplified DNA was visualized by electrophoresis on a 0.8% agarose gel in 1X TAE buffer (S1 File).

The purified product was sequenced commercially by Macrogen Inc. (Seoul, Korea) using primers, NL1 and NL4. The nucleotide sequences were compared using BLASTN [33]. The sequences with the highest scores were calculated by pairwise sequence similarity using a global alignment algorithm [34]. Phylogenetic and molecular evolution analyses were performed using Clustal Omega (https://www.ebi.ac.uk/jdispatcher/msa/clustalo). The neighbor-joining method uses the distance matrix of the alignment [35,36] for a phylogenetic tree based on the *26S* rDNA gene sequences of PSF strains and their phylogenetically related closest relatives. Bootstrap values (>50%) are shown at the branch nodes [32], using the UPGMA method and including an OUT-GROUP sequence for improved visualization. The PSF isolate sequences were submitted to NCBI (GenBank).

### Statistical analysis

The data were analyzed using analysis of variance (ANOVA), and the means were compared using the least significant difference test (LSD) at P ≤ 0.05. The treatments were arranged in a strip plot design for field crop traits, while a completely randomized design (CRD) was used for laboratory work. All statistical analyses were performed using Statistix 10 software [37].

## Results

### Fresh corn yield after fertilizer management

After growing fresh corn for two crops, the three fresh corn samples showed significant differences in ear length, ear diameter, and ear fresh weight (Table 1) at the harvest stage. Sweet corn provided the highest ear fresh weight of 6,853.80 kg ha^-1^ according to ear length and ear diameter based on plant characteristics, followed by pink and purple waxy corn. The application of organic and inorganic fertilizers resulted in a significant difference in fresh corn yield and biomass, except for ear diameter. The application of inorganic fertilizer (T4), BSF (T3), and vermicompost (T2) affected the fresh weight, ear length, 1000 seed weight, and harvest index (HI) of fresh corn. The maximum fresh weights were 6,291.30, 5,887.40, and 5,607.20 kg ha^-1^ respectively, in comparison to no fertilizer application. However, the interaction between variety and fertilizer showed no significant differences.

**Table 1:** Yield components of fresh corn grown under different fertilizer managements.

Treatment	Ear length (cm)	Ear diameter (cm)	Ear fresh weight (kg ha^-1^)	1000 seeds weight (g)	HI
**Corn varieties**					
Purple waxy (V1)	12.93 ± 1.37^b^	3.51 ± 0.74^b^	3,912.30 ± 903.28^c^	130.20 ± 28.76	0.30 ± 0.09
Pink waxy (V2)	11.60 ± 2.36^c^	4.20 ± 0.39^a^	5,520.80 ± 1,306.06^b^	140.50 ± 38.84	0.24 ± 0.03
Sweet (V3)	16.33 ± 1.73^a^	4.52 ± 0.33^a^	6,853.80 ± 1,771.72^a^	140.80 ± 49.94	0.19 ± 0.06
F-test (V)	**	**	**	ns	ns
CV (%)	7.63	10.09	15.44	62.56	53.13
**Fertilizer management**					
No fertilizer (T1)	12.01 ± 1.74^b^	3.79 ± 0.50	3,930.20 ± 990.59^b^	112.10 ± 42.53^b^	0.20 ± 0.08^b^
Vermicompost + 50% inorganic fertilizer (T2)	13.43 ± 3.20^ab^	4.33 ± 0.38	5,607.20 ± 1,832.72^a^	145.30 ± 36.78^a^	0.24 ± 0.08^ab^
BSF + 50% inorganic fertilizer (T3)	14.42 ± 2.70^a^	4.07 ± 0.81	5,887.40 ± 1,256.44^a^	147.60 ± 37.15^a^	0.25 ± 0.08^a^
Inorganic fertilizer (T4)	14.63 ± 2.60^a^	4.11 ± 0.83	6,291.30 ± 2,176.49^a^	143.60 ± 27.58^a^	0.27 ± 0.05^a^
F-test (T)	*	ns	**	*	*
CV (%)	10.04	10.09	15.89	13.02	14.44
**V x T**					
V1 x T1	11.72 ± 1.32	3.37 ± 0.71	2,925.20 ± 562.77	112.80 ± 44.93	0.24 ± 0.12
V1 x T2	12.30 ± 1.52	4.00 ± 0.40	3,836.40 ± 916.95	126.60 ± 25.15	0.29 ± 0.10
V1 x T3	14.24 ± 0.21	3.25 ± 1.04	4,591.10 ± 712.22	138.10 ± 20.72	0.34 ± 0.08
V1 x T4	13.47 ± 0.65	3.41 ± 0.87	4,296.60 ± 916.95	143.20 ± 25.42	0.31 ± 0.06
V2 x T1	10.59 ± 1.65	3.96 ± 0.22	4,354.90 ± 1,057.65	98.40 ± 10.60	0.22 ± 0.02
V2 x T2	11.29 ± 3.54	4.42 ± 0.38	6,030.20 ± 1,654.64	160.10 ± 28.65	0.25 ± 0.04
V2 x T3	11.92 ± 2.93	4.40 ± 0.14	6,029.90 ± 1,000.78	167.00 ± 31.27	0.24 ± 0.01
V2 x T4	12.63 ± 1.82	4.03 ± 0.62	5,668.40 ± 1,293.93	136.70 ± 8.74	0.24 ± 0.04
V3 x T1	13.72 ± 0.25	4.05 ± 0.18	4,510.30 ± 446.46	125.20 ± 67.56	0.16 ± 0.09
V3 x T2	16.69 ± 1.17	4.57 ± 0.17	6,954.90 ± 1,469.79	149.20 ± 55.47	0.17 ± 0.05
V3 x T3	17.11 ± 0.51	4.55 ± 0.13	7,041.30 ± 507.61	137.70 ± 57.17	0.18 ± 0.05
V3 x T4	17.80 ± 0.50	4.89 ± 0.09	8,908.80 ± 82.76	150.90 ± 46.56	0.25 ± 0.02
F-test (V x T)	ns	ns	ns	ns	ns
CV (%)	14.62	13.68	14.92	12.94	15.84

Mean ± SD; CV, coefficient of variation; ns, not significant difference; *, significant difference at P < 0.05; **, significant difference at P < 0.01. Different letters within the same column indicate significantly differences based on the Least Significant Difference (LSD) test at P < 0.05.

T1: no fertilizer (control); T2: 6,250 kg ha^-1^ of vermicompost + 95.31 kg N ha^-1 ^+ 23.44 kg P ha^-1 ^+ 23.44 kg K ha^-1^; T3: 6,250 kg ha^-1^ of black soldier fly (BSF) + 95.31 kg N ha^-1 ^+ 23.44 kg P ha^-1 ^+ 23.44 kg K ha^-1^; T4: 190.63 kg N ha^-1 ^+ 46.88 kg P ha^-1 ^+ 46.88 kg K ha^-1^.

### Soil chemical properties before and after growing three fresh corns

Before planting fresh corn, the soil samples showed significant differences in all chemical parameters, except N (Table 2). The soil was sandy loam, and the experimental site had acidic conditions. The purple waxy corn experimental site tended to have the highest chemical parameters, including pH, OM, available P, exchangeable Mg, Fe, Cu, Zn, and heavy metals, such as Cd, and Pb. The sweet corn experimental site tended to have the highest Ec, exchangeable Ca, Mn, and heavy metals, such as Cr and As. However, the total concentration of heavy metals in this experimental site was lower than in soils and toxic soils range for plant growth, except for Cd, which exceeded the normal range (0.35 mg kg^-1^) [38,39]. After growing three fresh corns for two crops, almost all soil chemical parameters showed significant differences between varieties and fertilizer application, except for total Pb between varieties (Table 3). The experimental soil used to cultivate the three fresh corns tended to increase the parameters of pH, available P, and Cu, and the chemical parameters of K, Ca, Mg, Mn, and Zn decreased. In the cultivation of sweet corn, and pink waxy corn, the chemical parameters of OM and total N tended to decrease compared to those of soil chemicals before cultivation. The application of vermicompost combined with inorganic fertilizer generally resulted in higher soil pH, total nitrogen (N), exchangeable potassium (K), manganese (Mn), and total cadmium (Cd). In contrast, the use of black soldier fly (BSF) mixed with inorganic fertilizer tended to increase organic matter (OM), electrical conductivity (EC), available phosphorus (P), exchangeable calcium (Ca), magnesium (Mg), iron (Fe), copper (Cu), as well as total chromium (Cr) and lead (Pb). Meanwhile, inorganic fertilizer alone was associated with higher EC and total arsenic (As). Additionally, the interaction between corn variety and fertilizer management revealed significant differences in soil chemical properties after the corn harvest. Vermicompost combined with inorganic fertilizer generally enhanced soil parameters such as pH, total N, exchangeable K, and total Cr. However, when purple waxy corn was grown with BSF mixed with inorganic fertilizer, soil fertility improved, particularly OM, available P, exchangeable Mg, and Fe. Furthermore, inorganic fertilizer alone affected soil pH, and some macro- and micronutrients showed a decrease. Soil pH is important for the dissolution, availability, and absorption of plant nutrients.

**Table 2:** Chemical characteristics of the experimental soil used before growing fresh corn.

Experimental soil	Chemical properties															
	pH	OM (%)	EC (dS m^-1^)	N (%)	P	K	Ca	Mg	Fe	Mn	Cu	Zn	Cr	As	Cd	Pb
					(mg kg^-1^)											
Purple waxy corn	5.06^a^	0.54^a^	0.03^c^	0.03	6.63^a^	77.93^a^	237.36^b^	45.53^a^	50.36^a^	4.25^b^	0.20^a^	1.23^a^	19.42^b^	1.04^b^	0.53^a^	3.45^a^
Pink waxy corn	4.75^c^	0.45^b^	0.03^b^	0.03	5.13^b^	77.33^a^	221.70^c^	42.97^b^	43.46^b^	4.29^b^	0.16^b^	0.61^b^	16.85^c^	0.93^b^	0.48^b^	3.24^ab^
Sweet corn	4.85^b^	0.53^a^	0.04^a^	0.03	3.13^c^	55.02^b^	358.32^a^	36.57^c^	39.82^c^	5.46^a^	0.17^b^	0.51^c^	21.73^a^	1.69^a^	0.52^ab^	3.05^b^
F-test	**	**	**	ns	**	**	**	**	**	**	**	**	**	**	*	**

Mean ± SD; ns, not significant; * = indicates significant difference at P < 0.05; ** = indicates significant difference at P < 0.01.

Numbers with different letters in each column indicate significant differences at P < 0.05, according to the least significant difference (LSD) test.

**Table 3:** Soil chemical characteristics after growing fresh corn.

Treatment	pH	OM (%)	EC (dS m^-1^)	N (%)	P	K	Ca	Mg	Fe	Mn	Cu	Zn	Cr	As	Cd	Pb
					(mg kg^-1^)											
**Corn varieties**																
Purple waxy (V1)	5.67^b^	0.67^a^	0.07^a^	0.05^a^	35.97^a^	117.66^a^	167.15^a^	29.74^a^	68.59^a^	2.93^a^	0.95^a^	0.07^b^	22.52^a^	2.07^a^	0.43^c^	3.89
Pink waxy (V2)	4.93^c^	0.53^b^	0.06^b^	0.03^c^	18.76^b^	92.76^b^	131.05^c^	28.84^a^	42.77^b^	2.92^a^	0.53^b^	0.11^a^	17.29^b^	1.22^b^	0.47^b^	3.85
Sweet (V3)	5.75^a^	0.50^c^	0.03^c^	0.04^b^	14.47^c^	93.16^b^	146.35^b^	24.50^b^	37.06^c^	2.48^b^	0.53^b^	0.05^c^	13.78^c^	1.07^c^	0.52^a^	3.79
F-test (V)	**	**	**	**	**	**	**	**	**	**	**	**	**	**	**	ns
CV (%)	0.71	1.57	3.16	8.03	0.23	1.12	1.90	4.98	1.64	1.04	4.52	4.11	0.71	6.10	0.86	5.40
**Fertilizer management**																
No fertilizer (T1)	5.59^b^	0.54^c^	0.03^c^	0.04^b^	6.30^d^	76.96^d^	127.05^d^	23.54^c^	48.89^c^	2.70^b^	0.75^a^	0.09^a^	16.65^d^	1.33^b^	0.50^a^	3.77^b^
Vermicompost + 50% inorganic fertilizer (T2)	5.87^a^	0.54^c^	0.04^b^	0.05^a^	23.64^b^	142.11^a^	154.10^b^	29.63^b^	45.24^d^	2.97^a^	0.74^a^	0.08^b^	17.50^c^	1.35^b^	0.49^a^	3.73^b^
BSF + 50% inorganic fertilizer (T3)	5.51^c^	0.60^a^	0.07^a^	0.04^b^	42.38^a^	104.86^b^	162.24^a^	38.20^a^	52.56^a^	2.74^b^	0.75^a^	0.08^b^	19.03^a^	1.27^c^	0.47^b^	4.01^a^
Inorganic fertilizer (T4)	4.82^d^	0.57^b^	0.07^a^	0.03^c^	19.93^c^	80.86^c^	149.34^c^	19.40^d^	51.21^b^	2.69^b^	0.44^b^	0.07^b^	18.27^b^	1.87^a^	0.43^c^	3.85^b^
F-test (T)	**	**	**	**	**	**	**	**	**	**	**	**	**	**	**	*
CV (%)	0.93	1.52	3.01	4.92	0.15	1.80	1.84	1.57	1.87	2.68	3.13	4.11	0.52	2.55	1.53	3.20
**V x T**																
V1 x T1	5.08^f^	0.60^c^	0.03^e^	0.03^d^	6.75^i^	62.60^i^	129.02^e^	24.49^e^	69.60^b^	2.10^i^	1.46^a^	0.07^e^	21.42^c^	1.95^b^	0.43^f^	4.04^ab^
V1 x T2	6.76^a^	0.67^b^	0.03^e^	0.08^a^	44.42^b^	211.97^a^	200.59^a^	30.73^d^	61.68^d^	3.23^b^	0.89^b^	0.08^d^	24.82^a^	2.03^b^	0.48^e^	3.97^ab^
V1 x T3	5.94^d^	0.72^a^	0.09^b^	0.05^b^	65.43^a^	101.74^d^	167.90^bc^	42.15^a^	78.51^a^	2.87^e^	0.89^b^	0.09^c^	24.45^a^	2.15^a^	0.45^f^	3.87^abc^
V1 x T4	4.89^g^	0.68^b^	0.12^a^	0.04^c^	27.28^e^	94.35^e^	171.08^b^	21.61^f^	64.55^c^	2.64^f^	0.59^f^	0.06^f^	19.38^e^	2.16^a^	0.35^h^	3.65^c^
V2 x T1	5.33^e^	0.50^f^	0.03^e^	0.03^d^	6.80^i^	71.89^h^	121.18^f^	24.87^e^	42.92^g^	2.62^f^	0.48^g^	0.14^a^	13.49^i^	1.11^d^	0.55^a^	3.85^bc^
V2 x T2	4.75^h^	0.49^f^	0.07^c^	0.03^d^	18.00^f^	96.66^e^	121.26^f^	32.24^d^	42.39^g^	3.09^c^	0.63^e^	0.11^b^	12.01^j^	0.90^f^	0.49^d^	3.43^d^
V2 x T3	4.64^i^	0.52^e^	0.09^b^	0.03^d^	32.23^c^	125.16^b^	116.86^g^	38.02^b^	44.96^e^	2.45^h^	0.58^f^	0.11^b^	22.61^b^	0.77^g^	0.42^g^	4.15^a^
V2 x T4	4.99^f^	0.60^c^	0.05^d^	0.03^d^	18.01^f^	77.34^g^	164.90^c^	20.24^f^	40.83^h^	3.52^a^	0.41^h^	0.09^c^	21.04^d^	2.08^ab^	0.42^g^	3.98^ab^
V3 x T1	6.36^b^	0.52^e^	0.02^f^	0.05^b^	5.36^j^	96.39^e^	130.95^e^	21.28^f^	34.14^j^	2.50^g^	0.30^i^	0.06^f^	15.05^g^	0.93^e^	0.52^b^	3.40^e^
V3 x T2	6.10^c^	0.48^g^	0.02^f^	0.03^d^	8.50^h^	117.69^c^	140.46^d^	25.93^e^	31.64^k^	2.59^g^	0.71^d^	0.06^f^	15.67^f^	1.12^c^	0.51^c^	3.78^b^
V3 x T3	5.95^d^	0.55^d^	0.03^e^	0.03^d^	29.50^d^	87.69^f^	201.96^a^	34.44^c^	38.33^i^	2.90^d^	0.80^c^	0.05^g^	10.02^k^	0.88^f^	0.54^a^	4.03^ab^
V3 x T4	4.59^i^	0.44^h^	0.05^d^	0.03^d^	14.50^g^	70.89^h^	112.04^g^	16.35^g^	44.11^f^	1.92^j^	0.32^i^	0.05^g^	14.40^h^	1.37^c^	0.52^b^	3.92^abc^
F-test (V x T)	**	**	**	**	**	**	**	**	**	**	**	**	**	**	**	**
CV (%)	1.08	1.29	3.16	4.92	0.27	2.42	1.36	2.97	1.66	2.02	3.81	4.11	1.49	6.81	2.76	2.98

Mean ± SD; CV, coefficient of variation; ns, not significant difference; *, significant difference at P < 0.05; **, significant difference at P < 0.01.

Different letters within the same column indicate significantly differences based on the Least Significant Difference (LSD) test at P < 0.05.

T1: no fertilizer (control); T2: 6,250 kg ha^-1^ of vermicompost + 95.31 kg N ha^-1 ^+ 23.44 kg P ha^-1 ^+ 23.44 kg K ha^-1^; T3: 6,250 kg ha^-1^ of black soldier fly (BSF) + 95.31 kg ha^-1 ^+ 23.44 kg P ha^-1 ^+ 23.44 kg K ha^-1^; T4: 190.63 kg N ha^-1 ^+ 46.88 kg P ha^-1 ^+ 46.88 kg K ha^-1^.

### The isolation of PSM from bulk soil after growing fresh corn

**Fig 1:**
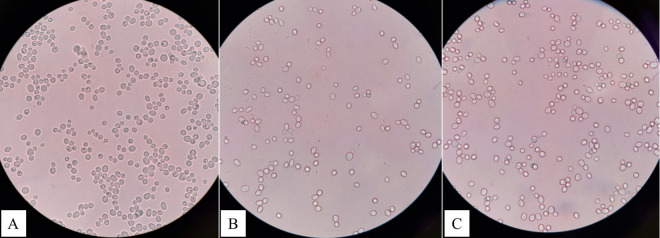
Phosphate solubilizing microorganisms with clear halozone produced by, (A) Sw-T1, (B) Pu-T2, and (C) Pu-T4 isolates.

Phosphate-solubilizing microorganisms were isolated from the soil samples before and after the growth of fresh corn using NBRIP growth media. The target PSM after growing fresh corn from the second crop produced a clear halo zone around the colony on NBRIP solid media (Fig 1) compared with the isolation of PSM from bulk soil before and after growing fresh corn from the first crop. There are 3 isolates of PSM were found, one isolate was found from sweet corn with no fertilizer application (Sw-T1) (Fig 1A), and two PSM isolates were found from purple waxy under the application of the vermicompost mix with inorganic fertilizer (Pu-T2) (Fig 1B) and the application of inorganic fertilizer (Pu-T4) (Fig 1C) (Table 4). In addition, the highest PSM population was found in purple waxy with 3.87 × 10^8^ and 5.67 × 10^8^ CFU l^-1^ respectively.

**Table 4:** The density of PSM isolates from bulk soil after growing fresh corn under different fertilizers application.

PSMs isolates	The Population of PSMs × 10^8^ (CFU) l^-1^
Sw-T1	0.17 ± 0.003^b^
Pu-T2	3.87 ± 0.66^a^
Pu-T4	5.67 ± 0.98^a^
F-test	**
CV (%)	39.15

Mean ± SD; CV, coefficient of variation; **, indicates significant difference at P < 0.01.

Different letters within the same column indicate significantly differences based on the least significant difference (LSD) test at P < 0.05.

Sw-T1 = Sweet corn + no fertilizer (control); Pu-T2 = Purple waxy + 6,250 kg ha^-1^ of vermicompost + 95.31 kg N ha^-1 ^+ 23.44 kg P ha^-1 ^+ 23.44 kg K ha^-1^; Pu-T4 = Purple waxy + 190.63 kg N ha^-1 ^+ 46.88 kg P ha^-1 ^+ 46.88 kg K ha^-1^.

### Phosphate-solubilizing microorganism effectiveness of tested P solubilization

The ability of PSM to dissolve P derived from AlPO_4_, Ca_3_(PO_4_)_2_, and FePO_4_ is presented in (Table 5). Among the three isolates, Pu-T4 and Sw-T1 showed the highest concentrations of Ca_3_(PO_4_)_2_ at 622.55 mg l^-1^ and 616.95 mg l^-1^ respectively. However, the ability to dissolve unavailable forms such as AlPO_4_ and FePO_4_ in sandy soils with low pH was not significantly different among the three PSM isolates.

**Table 5:** Phosphate-solubilizing effectiveness of tested PSM isolates from bulk soil after growing fresh corn under different fertilizers application.

PSM Isolates	Solubilized Phosphate (mg l^-1^)		
	AlPO_4_	Ca_3_(PO_4_)_2_	FePO_4_
Sw-T1	618.81 ± 5.88	616.95 ± 16.84^a^	635.77 ± 10.30
Pu-T2	607.86 ± 14.26	535.63 ± 19.44^b^	603.59 ± 5.48
Pu-T4	629.76 ± 21.66	622.55 ± 12.11^a^	611.07 ± 12.99
F-test	ns	*	ns
CV (%)	4.30	4.80	2.83

Mean ± SD; CV, coefficient of variation; ns, not significant; *, indicates significant difference at P < 0.05.

Different letters within the same column indicate significantly differences based on the least significant difference (LSD) test at P < 0.05.

Sw-T1 = Sweet corn + no fertilizer (control); Pu-T2 = Purple waxy + 6,250 kg ha^-1^ of vermicompost + 95.31 kg N ha^-1 ^+ 23.44 kg P ha^-1 ^+ 23.44 kg K ha^-1^; Pu-T4 = Purple waxy + 190.63 kg N ha^-1 ^+ 46.88 kg P ha^-1 ^+ 46.88 kg K ha^-1^.

### Production of indole-3-acetic acid by PSM

The selected plant growth-promoting PSM was evaluated by analyzing IAA (Table 6). The two PSM isolated showed the ability to produce IAA, indicating that these strains could utilize l-tryptophan as a precursor for plant growth. Pu-T2 isolate showed the highest IAA production (562.81 mg l^-1^) followed by Pu-T4 isolate (462.81 mg l^-1^).

**Table 6:** IAA production of PSM isolates from bulk soil after growing fresh corn under different fertilizers application.

PSMs Isolates	IAA Production (mg l^-1^)
Pu-T2	562.81 ± 1.73^a^
Pu-T4	462.81 ± 5.81^b^
F-test	**
CV (%)	1.45

Mean ± SD; CV, coefficient of variation; **, indicates significant difference at P < 0.01.

Different letters within the same column indicate significantly differences based on the least significant difference (LSD) test at P ≤ 0.05.

Sw-T1 = Sweet corn + no fertilizer (control); Pu-T2 = Purple waxy + 6,250 kg ha^-1^ of vermicompost + 95.31 kg N ha^-1 ^+ 23.44 kg P ha^-1 ^+ 23.44 kg K ha^-1^; Pu-T4 = Purple waxy + 190.63 kg N ha^-1 ^+ 46.88 kg P ha^-1 ^+ 46.88 kg K ha^-1^.

### Phosphate-solubilizing fungi strains characterization and genetic identification

**Fig 2:**
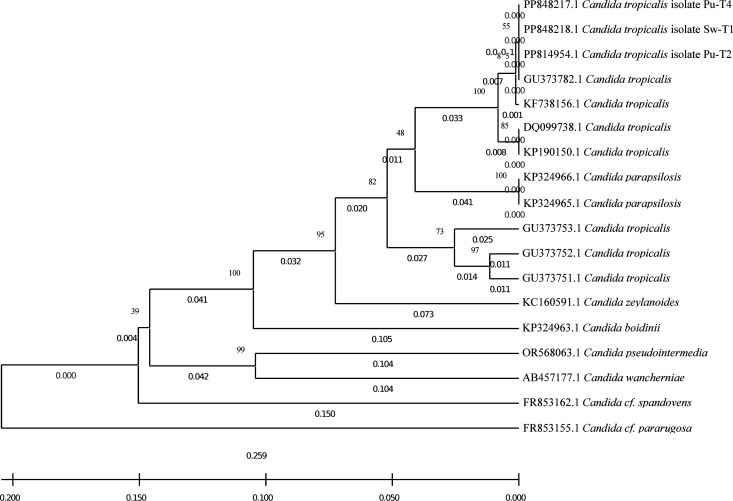
Phylogenetic tree of PSF isolates from bulk soil of fresh corn by *26S* rDNA sequencing and the deposited sequence in NCBI.

PSF isolates were characterized by DNA extraction, PCR amplification, and sequencing, and the sequences were aligned using a BLASTN search. The results showed that Sw-T1, Pu-T2, and Pu-T4 had 100.00% similarity with the type strain of *Candida tropicalis* ATCC 750T (NG_054834) (S1 File). These PSF isolate sequences were submitted to NCBI with the accession numbers SUB14477274 Sw-T1 PP848218, SUB14459899 Pu-T2 PP814954, and SUB14477270 Pu-T4 PP848217 (S2 File). Multiple sequence alignments with other *Candida* isolates deposited in GENBANK included 10 *C. tropicalis*, 2 *C. parapsilosis*, *C. boidinii*, *C. zeylanoides*, *C. santamariae*, *C. cf. pararugosa*, 2 *C. cf. spandovensis*, *C. intermedia,* and *C. pseudointermedia* as shown in Fig 2.

## Discussion

### Effect of organic and inorganic fertilizers on fresh corn yield

Under field conditions, sweet corn showed the highest ear yield compared with pink and purple waxy corn. Differences in genetic traits and phenotypic expression of corn varieties may also play a role in different growth habits under environmental conditions. The N from organic and inorganic fertilizers (T2-T4) may promote photosynthetic activity and improve fresh corn yield production in comparison to no fertilizer (T1) application. In addition, pink waxy corn appears to be interesting because it provides a better ear yield than purple waxy corn under acid sandy soil. Applying 6,250 kg ha^-1^ of BSF combined with 50% of inorganic fertilizer (T3) results in yields comparable to those of inorganic fertilizer (T4), with a significant difference from the no fertilizer treatment (T1). Similarly, Shirkhani et al. [40] reported that vermicompost 6,000 kg ha^-1^ mixed with 100% inorganic recommended fertilizer application tended to increase the grain yield of corn. Tufa [18] found that the combination of vermicompost at 7,500 kg ha^-1^ with inorganic NPSZnB fertilizer at a rate of 150 kg ha^-1^ yielded the highest sweet corn yield of 8,003 kg ha^-1^. Therefore, the application of organic fertilizers significantly reduces the consumption of inorganic fertilizers and adverse environmental effects [41]. In addition, the application of BSF fertilizer at 7,500 kg ha^-1^ produced the highest corn grain yields during the short (5,400 kg ha^-1^) and long rain seasons (7,200 kg ha^-1^), which were higher than the highest grain yields obtained using commercial organic fertilizer [42].

### Organic phosphate improves soil chemical properties after fresh corn growing

After growing three fresh corn varieties for the two crops, all soil chemical parameters showed a significant difference between varieties, fertilizer application, and interaction. The experimental soil used to cultivate purple waxy corn tended to have the highest OM, EC, total N, available P, exchangeable K, Fe, total Cr, and As contents. In addition, inorganic fertilizers tend to reduce the soil pH and some nutrients in the long term. The results showed that exchangeable Zn and total Cr tended to decrease after growing the three fresh corn crops under fertilizer management in both crops. The use of organic matter for crop growth and soil fertility involves the decomposition of plant residues. Soil organic matter determines Cr bioavailability in soil through oxidation, adsorption, or desorption [43]. It binds metals in the soil and acts as a transporter of Cr and several other heavy metals [44]. Soil OM drives microbial growth and indirectly stimulates biological reduction of Cr in soil [45]. A decrease in soil pH causes the mobilization and release of Cr, whereas an increase in soil pH leads to the formation of Cr in the soil [46]. Over time, Cr under no fertilizer (T1) and fertilizer management (T2-T4) may decrease in concentration in the soil solution by chemical weathering processes, such as dissolution, precipitation, and ion exchange. The dissolution of chromium-bearing minerals and subsequent leaching may decrease soil Cr levels, especially in areas with high rainfall or acidic soil conditions [47]. Moreover, inorganic fertilizers containing ammonium ions can acidify the soil, leading to a decrease in soil pH and promoting the retention of Cr in its less soluble forms, thereby decreasing its availability for plant uptake and leaching [48]. A decrease in soil Zn levels can result from a combination of leaching, chemical reactions, microbial activity, and plant uptake processes. Zn is considered the most required micronutrient [49]for structuring and enzyme functioning; and is involved in the regulation of various biochemical and physiological processes [50].

At the same time, the decrease in soil As levels after growing sweet corn compared with waxy corn can result from differences in root characteristics, physiological traits, genetic variations, rhizosphere interactions, and environmental factors [51]. Roba [52] reported that inorganic fertilizers can rapidly influence soil chemistry. Nitrogen is rapidly available for plant uptake, whereas P and K can affect soil chemistry within a few weeks to months. Organic fertilizers have a slower effect on soil chemistry than inorganic fertilizers. Organic matter requires several months to years to decompose and release nutrients. The full impact on the soil structure, nutrient content, and pH levels may be observed over a longer period. Organic amendments can gradually increase soil pH, but this process may take several months to a few years to achieve significant changes, especially in highly acidic soils. Moreover, changing soil chemical properties after growing fresh corn for the two crops may occur because of heavy rain during crop growth. Combining inorganic and organic fertilizers can provide more immediate benefits while also contributing to long-term soil improvement, as inorganic fertilizers can supply quick nutrient boosts, and organic fertilizers enhance soil structure and fertility over time.

### The diversity of PSF from bulk soil after growing fresh corn

After harvesting the corn yield, soil samples were collected for PSF isolation. Fertilizer management did not show an effect on PSF isolates because one PSF isolate from the bulk soil of sweet corn was found under no fertilizer application (Sw-T1). Two PSF isolates were found in the bulk soil of purple waxy grown under vermicompost mixed with inorganic fertilizer (Pu-T2) and inorganic fertilizer (Pu-T4). Similar to Enebe and Babalola [53] and Geisseler et al. [54], both inorganic and organic fertilizers affected the corn rhizosphere microbes, with organic amendments promoting the largest microbial community. In contrast, inorganic fertilizer alone did not significantly increase soil microbial abundance. Rather, the abundance of individual bacterial or fungal species was more sensitive and primarily linked to changes in soil chemical properties caused by the application of inorganic or organic fertilizers. Both types of fertilizers can directly promote the growth of specific microbial populations [55]. However, inorganic fertilizer management does not significantly influence the richness and diversity of bacteria and fungi [56]. In several studies, N fertilizer led to a 15.1% increase in microbial biomass compared to no fertilizer plots [54,57]. Zhang et al. [58] reported that inorganic fertilizer at 60 kg N ha^-1^ negatively affected the microbial community structure and abundance in agricultural soils. However, plant growth stages may be relevant to root exudate in the rhizosphere; fungi were found more in root plants at the seedling stage than at the silking stage [59].

Moreover, PSF was found to be only 0.1–0.5% in soil [12]. The difference in PSF density may be due to variations in rhizosphere microbial communities among various plant species [60]. Our PSF results, were not observed in the bulk soil of pink waxy corn. Phosphate-solubilizing fungi were also not found in the bulk soil before or after growing fresh corn in the first crop. The soil may lack specific nutrients or conditions (organic matter) for microorganisms to thrive. Growing corn alters the soil environment by increasing the amount of organic materials and nutrients that promote PSF growth. Soil moisture, pH, and temperature may change due to corn cultivation, creating more favorable conditions for PSF development. Corn roots release exudates (organic acids, sugars, and other compounds) that can stimulate PSF growth. These exudates serve a food sources for soil microorganisms, promoting their activity and population growth. Phosphate-solubilizing fungi may establish a symbiotic relationship with corn roots, helping the plant to acquire phosphorus while benefiting from root exudates. Before grow, the absence of these roots limits the presence [61].

### The ability of PSF to dissolve P and indole acetic acid production

**Fig 3:**
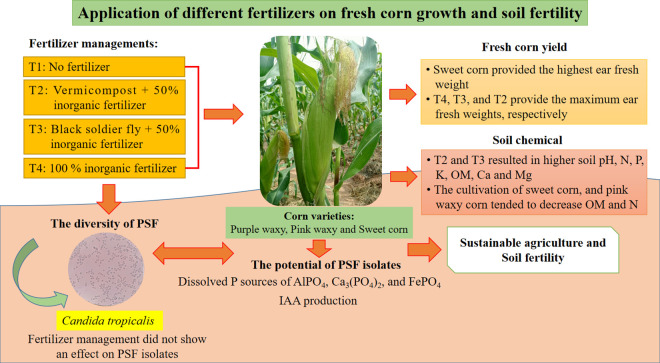
Conceptual diagram shows the effect of different fertilizers on fresh corn growth and soil fertility.

The use of organic phosphate efforts to increase available P and nitrogen (N) nutrients status for crop growth [62], as our conceptual diagram shows the effect of different fertilizers on fresh corn growth and soil fertility (Fig 3). Also, biofertilizers are carrier media that maintain the necessity of phosphate-solubilizing microorganisms (PSM). Some PSM (bacteria, fungi, and actinomycetes) that live freely in the soil and biofertilizers can extract phosphate from insoluble to available forms through the secretion of organic acids [63–65]. Three PSF isolates (Sw-T1, Pu-T2, and Pu-T4) from bulk soil after growing sweet and purple waxy corn with different fertilizer applications for two crops showed 100.00% identical similarity with the type strain of *Candida tropicalis* ATCC 750T (NG_054834). Similar to Sarabia et al. [66], fungi were isolated from rhizosphere soil samples during the vegetative, flowering, and senescence growth phases of six corn in Mexico. *Ascomycetous* was dominant, and the most prevalent species were *Candida railenensis* and *Meyerozyma guiliermondii*. Recent research highlights that environmental variables such as plant genotype, soil pH, and organic carbon significantly influence PSF diversity and potential [67]. Organic fertilizer increases the abundance of beneficial microbes and improves the enzymatic activities related to nutrient cycling, contributing to the natural recruitment of phosphate solubilizers [68].

Phosphate-solubilizing fungi from the bulk soil of purple waxy and sweet corn were dissolved P sources of AlPO_4_, Ca_3_(PO_4_)_2_, and FePO_4_ were not significantly different from AlPO_4_ and FePO_4_ sources. The P sources of AlPO_4_ and FePO_4_ are the main issues in fixing the available P in acidic soils. Similarly, Quan et al. [69] reported that the greatest effect of dissolving P was found in a medium containing AlPO_4_, FePO_4_, and Ca_3_(PO_4_)_2_, by PSF from the chickpea root rhizosphere. In addition, PSF from the bamboo root rhizosphere had higher dissolved P from AlPO_4_ and FePO_4_ than Ca_3_(PO_4_)_2_ [70]. PSF was isolated from peat soil and showed the greatest effect of dissolving P sources of Ca_3_(PO_4_)_2_, FePO_4_, and AlPO_4_, according to the complex structure of AlPO_4_ and FePO_4_ [71]. Fertilizer application, was not affected by dissolved P from AlPO_4_ and FePO_4_ among all PSF. Previous research has demonstrated that inorganic fertilizers alter microbial population abundance and promote their growth to provide essential nutrients. *Knufia petricola* and *Zygomycetes* were found only in the inorganic fertilizer application [72], while *Firmicutes*, *Proteobacteria*, and *Zygomycota* well under organic fertilizer application [56]. Additionally, PSF strains like *Penicillium* and *Aspergillus* spp. exhibit strain-specific efficiency in solubilizing rock phosphate under varying pH conditions, suggesting that isolate selection is crucial for biofertilizer development [73]. These findings are consistent with recent studies on the importance of PSF-produced organic acids in solubilizing Fe and Al-bound phosphates. According to Pang et al. [74], organic acids chelate the metal cations, resulting in the release of soluble phosphate from insoluble compounds like AlPO₄ and FePO_4_. PSF activity was more efficient under acidic conditions, supporting that PSF selection for acid soils is critical for improve phosphorus bioavailability.

Regarding the ability of PSF to produce IAA, only two PSF isolates from the bulk soil of purple waxy corn produced IAA. The highest concentration of IAA was produced in the Pu-T2 isolate under the vermicompost mix with inorganic fertilizer at 562.81 mg l^-1^. *Aspergillus niger* was isolated from peat soils and produced IAA at 2,363 mg l^-1^ [71]. Differences in regulatory mechanisms among PSF can contribute to variations in their ability to produce IAA and dissolve P. Some PSF can produce high levels of IAA, whereas others may produce lower amounts or may not produce it at all. Meanwhile, some isolates may dissolve more P, but produce lower quantities or do not produce IAA. Our results showed that Sw-T1 isolates from the bulk soil of sweet corn had the highest significant difference in dissolving P, but could not produce IAA. Current studies indicate that the production of IAA by PSF is influenced by environmental factors such as tryptophan availability, carbon source, and pH [75]. IAA promotes root development, improving nutrient absorption, and increasing stress resistance, which supports higher crop yields and better soil health in sustainable agriculture [76]. According to Alemneh et al. [77], IAA may affect the activity of genes involved in producing organic acids, which are crucial for dissolving phosphate. Albertini et al. [78] found that environmental and nutritional factors such as nitrogen availability and pH strongly affect IAA production in rhizospheric yeast. These findings support the hypothesis that PSF capable of phosphate solubilization and IAA production may be more effective in promoting plant growth and nutrient uptake. Annadurai et al. [79] reported that *C. tropicalis* can also interact with other microorganisms to promote plant growth. When *C. tropicalis* isolated from bean roots was inoculated alongside the bacterium *Rhizobium* sp., it enhanced nodulation and nutrient absorption to improve plant growth. *C. tropicalis* boosts nodulation through its distinctive metabolites, including indole compounds, tryptophan, phenolic compounds, and α-D-galactopyranoside. Genomic studies have revealed that *C. tropicalis* possesses multiple phosphate transporter genes and enzymes like phosphatases, which have high solubilization capacity [80]. Furthermore, multilocus sequence typing and whole genome sequencing analyses have revealed high genetic diversity among *Candida tropicalis* isolates from different geographic regions, indicating potential ecological adaptation and niche specialization [81].

## Conclusions

This study demonstrates that reducing the use of inorganic fertilizer by 50% following GAP standards, along with the application of black soldier fly (T3) and vermicompost (T2), maintained the ear yield of the three fresh corn varieties, mainly sweet corn, at 6,853.80 kg ha^-1^. After growing three fresh corn crops for two crops, most soil chemical parameters tended to increase under the organic fertilizer mix with an inorganic fertilizer compared to 100% inorganic fertilizer application. However, exchangeable Zn and total Cr decreased after growing three fresh corn plants under fertilizer application. Application of fertilizer did not affect the diversity of phosphate-solubilizing fungi (PSF). Three PSF isolates were collected from the bulk soil after growing purple waxy and sweet corn for two crops belonging to Candida tropicalis, which is part of the genus Candida compared to the bulk soil before growing fresh corn. The potential for solubilizing AlPO_4_, and FePO_4_ was not significantly different between the PSF isolates. Only two PSF isolates from the bulk soil of purple waxy corn produced IAA hormone at 462.81–562.81 mg l^-1^.

## Supporting information

S1 File Lab protocol. (DOCX)

S2 File Link of PSF sequences deposited in NCBI. (DOC)
