# Supplementary material for: Changes in the bulk soil after fresh corn grown with organic and inorganic fertilizer application
Source: PLoS One. 2025 Jul 10;20(7):e0326730. doi: 10.1371/journal.pone.0326730 (PMC12244754; doi:10.1371/journal.pone.0326730)
Supplement: S1 File — (DOCX) [file pone.0326730.s001.docx]

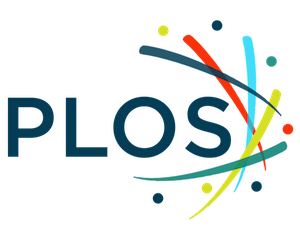


Changes in the bulk soil after fresh corn grown with organic and inorganic fertilizer application

Riri Dayang Sari Risman^1^, Kiriya Sungthongwises^1^,* Supanath Kanjanawattanawong^2^

1 Agronomy section, Faculty of Agriculture, Khon Kaen University, Khon Kaen, Thailand

2 Horticulture section, Faculty of Agriculture, Khon Kaen University, Khon Kaen, Thailand

## Associated content

Minimum include DOI for protocol on protocols.io

# Abstract

The effects of different fertilizer applications on crop growth, soil health, and microbial communities are critical for sustainable agriculture. Positive interactions between crop roots and their associated microbiomes are essential to improve nutrient availability and promote plant growth. Therefore, this study aimed to investigate the changes in bulk soil chemical properties and diversity of phosphate-solubilizing microorganisms after growing three fresh corn plants under the application of vermicompost, black soldier flies, and inorganic fertilizers. Fresh corn yield and soil samples were collected from purple waxy, pink waxy, and sweet corn grown under field conditions. The capacity to solubilize mineral phosphate and indole acetic acid was also determined using a spectrophotometer. The results showed that organic and inorganic fertilizers can maintain the ear-fresh weight of the three fresh corn varieties and tend to increase some soil chemicals after growth. Application of inorganic fertilizer and black soldier flies mixed with inorganic fertilizer resulted in the highest ear fresh weight, with 6,291.30 and 5,887.40 kg ha^-1^, respectively. Moreover, the soil pH, available phosphorus, and copper tended to increase, whereas zinc and chromium decreased. However, fertilizer management did not affect the diversity of the phosphate-solubilizing microorganisms. In addition, the three phosphate-solubilizing fungal isolates were similar to the type strain of *Candida tropicalis*. The phosphate-solubilizing fungi isolate potentials were not significantly different in AlPO_4_, and FePO_4_ solubilizing. Only two PSF isolates from purple waxy produced IAA hormone between 462.81-562.81 mg l^-1^.

# Steps

# Isolation of PSM isolated DNA for PCR and Identification of PSF were conducted at a commercial laboratory, Thailand Bioresource Research Center (TBRC), in Bangkok, Thailand.

Methods for DNA isolation and amplification of the D1/D2 domain of the LSU rRNA gene

1. Isolation of DNA for Polymerase Chain Reaction (PCR)
2. Polymerase Chain Reaction (PCR) for D1/D2 domain of *26S* rDNA
3. D1/D2 domain of *26S* rDNA sequencing
4. BLAST analysis
5. Expected results

1. Isolation of DNA for Polymerase Chain Reaction (PCR)

CITATION

Manitis, T., E.F. Fritsch, and J. Sambrook. 1982. Molecular cloning: a laboratory manual. Cold Spring Harbor laboratory: Cold Spring Harbor, New York U.S.A.

LINK

https://doi.org/10.1002/jobm.19840240107

Isolation of DNA was carried out by boiling of cells with lysis buffer according to the methods of Manitis et al. (1982) with slight modification. A loopful of yeast cells was transferred to 1.5 ml Eppendorf tube. The 100 l of lysis buffer was added. Cell suspensions were boiled in water bath or metal block bath for 15 min. After boiling, 100 l of 2.5 M potassium acetate (pH 7.5) was added and placed on ice for 1 hr, and centrifuged at 14,500 rpm for 5 min. Supernatant was extracted twice with 100 l of chloroform:isoamyl alcohol (24:1 v/v). DNA was precipitated with isopropanol, placed at 20°C for 10 min and centrifuged at 14,500 rpm for 15 min. DNA pellet was rinsed with 70% and 90% ethanol and then dried up (15-30 min at room temperature). The dried DNA was dissolved in 30 l nanopure water.

2. Polymerase Chain Reaction (PCR) for D1/D2 domain of *26S* rDNA

CITATION

Kurtzman CP, Robnett CJ (1998). Identification and phylogeny of ascomycetous yeasts from analysis of nuclear large subunit (*26S*) ribosomal DNA partial sequences. Antonie van Leeuwenhoek. 73: 331-371.

LINK

DOI: 10.1023/a:1001761008817

The divergent D1/D2 domain of *26S* rDNA was amplified with primers NL-1 (5’- GCA TAT CAA TAA GCG GAG GAA AAG-3’) and NL4 (5’-GGT CCG TGT TTC AAG ACG G-3’) (Kurtzman and Robnett, 1998). The specific make and model of the PCR thermal cycler is Biorad model T100. Amplification was carried out in 100 l reaction mixture conditioning 100 ng of genomic DNA, 5 U/µl of Taq polymerase, 2.5 mM of each dNTP, 10 pM of each primer, 10X Taq buffer and 25 mM MgCl_2_

The reaction was pre-denatured at 94ºC for 5 min, then repeated for 30 PCR cycles with denaturation at 94ºC for 1 min, annealing at 52ºC for 1.30 min and extension at 72ºC for 2.30 min and then followed by the final extension at 72ºC for 10 min. The PCR product was checked by agarose gel electrophoresis and purified by using the GenepHlowTM Gek/PCR Kit (Geneaid Biotech Ltd., Taiwan). Visualization of the purified amplified DNA was performed by electrophoresis using 0.8% agarose gel in 1X TAE buffer.

3. D1/D2 domain of *26S* rDNA sequencing

The purified product was sequenced commercially by Macrogen Inc. (Seoul, Korea) for sequencing with primers, NL1 and NL4.

4. BLAST analysis

The sequences were compared using a BLASTN search (http://www.ncbi.nlm.nih.gov/blast).

5. Expected results

Nucleotide sequence of D1/D2 region of the large subunit rRNA gene

>Pu-T2 (NL-1 and NL-4)_578 nucleotides

AAACCAACAGGGATTGCCTTAGTAGCGGCGAGTGAAGCGGCAAAAGCTCAAATTTGAAATCTGGCTCTTTCAGAGTCCGAGTTGTAATTTGAAGAAGGTATCTTTGGGTCTGGCTCTTGTCTATGTTTCTTGGAACAGAACGTCACAGAGGGTGAGAATCCCGTGCGATGAGATGATCCAGGCCTATGTAAAGTTCCTTCGAAGAGTCGAGTTGTTTGGGAATGCAGCTCTAAGTGGGTGGTAAATTCCATCTAAAGCTAAATATTGGCGAGAGACCGATAGCGAACAAGTACAGTGATGGAAAGATGAAAAGAACTTTGAAAAGAGAGTGAAAAAGTACGTGAAATTGTTGAAAGGGAAGGGCTTGAGATCAGACTTGGTATTTTGTATGTTACTTCTTCGGGGGTGGCCTCTACAGTTTATCGGGCCAGCATCAGTTTGGGCGGTAGGAGAATTGCGTTGGAATGTGGCACGGCTTCGGTTGTGTGTTATAGCCTTCGTCGATACTGCCAGCCTAGACTGAGGACTGCGGTTTATACCTAGGATGTTGGCATAATGATCTTAAGTCGCCCGTCTTG

= The strain Pu-T2 was identified as *Candida tropicalis*.

>Pu-T4 (NL-1 and NL-4)_578 nucleotides

AAACCAACAGGGATTGCCTTAGTAGCGGCGAGTGAAGCGGCAAAAGCTCAAATTTGAAATCTGGCTCTTTCAGAGTCCGAGTTGTAATTTGAAGAAGGTATCTTTGGGTCTGGCTCTTGTCTATGTTTCTTGGAACAGAACGTCACAGAGGGTGAGAATCCCGTGCGATGAGATGATCCAGGCCTATGTAAAGTTCCTTCGAAGAGTCGAGTTGTTTGGGAATGCAGCTCTAAGTGGGTGGTAAATTCCATCTAAAGCTAAATATTGGCGAGAGACCGATAGCGAACAAGTACAGTGATGGAAAGATGAAAAGAACTTTGAAAAGAGAGTGAAAAAGTACGTGAAATTGTTGAAAGGGAAGGGCTTGAGATCAGACTTGGTATTTTGTATGTTACTTCTTCGGGGGTGGCCTCTACAGTTTATCGGGCCAGCATCAGTTTGGGCGGTAGGAGAATTGCGTTGGAATGTGGCACGGCTTCGGTTGTGTGTTATAGCCTTCGTCGATACTGCCAGCCTAGACTGAGGACTGCGGTTTATACCTAGGATGTTGGCATAATGATCTTAAGTCGCCCGTCTTG

= The strain Pu-T4 was identified as *Candida tropicalis*.

>Sw-T1 (NL-1 and NL-4)_578 nucleotides

AAACCAACAGGGATTGCCTTAGTAGCGGCGAGTGAAGCGGCAAAAGCTCAAATTTGAAATCTGGCTCTTTCAGAGTCCGAGTTGTAATTTGAAGAAGGTATCTTTGGGTCTGGCTCTTGTCTATGTTTCTTGGAACAGAACGTCACAGAGGGTGAGAATCCCGTGCGATGAGATGATCCAGGCCTATGTAAAGTTCCTTCGAAGAGTCGAGTTGTTTGGGAATGCAGCTCTAAGTGGGTGGTAAATTCCATCTAAAGCTAAATATTGGCGAGAGACCGATAGCGAACAAGTACAGTGATGGAAAGATGAAAAGAACTTTGAAAAGAGAGTGAAAAAGTACGTGAAATTGTTGAAAGGGAAGGGCTTGAGATCAGACTTGGTATTTTGTATGTTACTTCTTCGGGGGTGGCCTCTACAGTTTATCGGGCCAGCATCAGTTTGGGCGGTAGGAGAATTGCGTTGGAATGTGGCACGGCTTCGGTTGTGTGTTATAGCCTTCGTCGATACTGCCAGCCTAGACTGAGGACTGCGGTTTATACCTAGGATGTTGGCATAATGATCTTAAGTCGCCCGTCTTG

= The strain Sw-T1 was identified as *Candida tropicalis*.

Sequence similarity

Sequence similarity of strain Pu-T2, Pu-T4 and Sw-T1

**>NG_054834.1 *Candida tropicalis* ATCC 750T (Type strain)** *28S* rRNA, partial sequence; from TYPE material

Sequence ID: Query_4881823Length: 865Number of Matches: 1

Range 1: 6 to 583GraphicsNext MatchPrevious Match

Alignment statistics for match #1

Score Expect Identities Gaps Strand

1068 bits (578) 0.0 578/578 (100%) 0/578 (0%) Plus/Plus

**Query = Pu-T2, Pu-T4 and Sw-T1**

**Sbject = NG_054834 *Candida tropicalis* ATCC 750T (Type strain)**

Query 1 AAACCAACAGGGATTGCCTTAGTAGCGGCGAGTGAAGCGGCAAAAGCTCAAATTTGAAAT 60

||||||||||||||||||||||||||||||||||||||||||||||||||||||||||||

Sbjct 6 AAACCAACAGGGATTGCCTTAGTAGCGGCGAGTGAAGCGGCAAAAGCTCAAATTTGAAAT 65

Query 61 CTGGCTCTTTCAGAGTCCGAGTTGTAATTTGAAGAAGGTATCTTTGGGTCTGGCTCTTGT 120

||||||||||||||||||||||||||||||||||||||||||||||||||||||||||||

Sbjct 66 CTGGCTCTTTCAGAGTCCGAGTTGTAATTTGAAGAAGGTATCTTTGGGTCTGGCTCTTGT 125

Query 121 CTATGTTTCTTGGAACAGAACGTCACAGAGGGTGAGAATCCCGTGCGATGAGATGATCCA 180

||||||||||||||||||||||||||||||||||||||||||||||||||||||||||||

Sbjct 126 CTATGTTTCTTGGAACAGAACGTCACAGAGGGTGAGAATCCCGTGCGATGAGATGATCCA 185

Query 181 GGCCTATGTAAAGTTCCTTCGAAGAGTCGAGTTGTTTGGGAATGCAGCTCTAAGTGGGTG 240

||||||||||||||||||||||||||||||||||||||||||||||||||||||||||||

Sbjct 186 GGCCTATGTAAAGTTCCTTCGAAGAGTCGAGTTGTTTGGGAATGCAGCTCTAAGTGGGTG 245

Query 241 GTAAATTCCATCTAAAGCTAAATATTGGCGAGAGACCGATAGCGAACAAGTACAGTGATG 300

||||||||||||||||||||||||||||||||||||||||||||||||||||||||||||

Sbjct 246 GTAAATTCCATCTAAAGCTAAATATTGGCGAGAGACCGATAGCGAACAAGTACAGTGATG 305

Query 301 GAAAGATGAAAAGAACTTTGAAAAGAGAGTGAAAAAGTACGTGAAATTGTTGAAAGGGAA 360

||||||||||||||||||||||||||||||||||||||||||||||||||||||||||||

Sbjct 306 GAAAGATGAAAAGAACTTTGAAAAGAGAGTGAAAAAGTACGTGAAATTGTTGAAAGGGAA 365

Query 361 GGGCTTGAGATCAGACTTGGTATTTTGTATGTTACTTCTTCGGGGGTGGCCTCTACAGTT 420

||||||||||||||||||||||||||||||||||||||||||||||||||||||||||||

Sbjct 366 GGGCTTGAGATCAGACTTGGTATTTTGTATGTTACTTCTTCGGGGGTGGCCTCTACAGTT 425

Query 421 TATCGGGCCAGCATCAGTTTGGGCGGTAGGAGAATTGCGTTGGAATGTGGCACGGCTTCG 480

||||||||||||||||||||||||||||||||||||||||||||||||||||||||||||

Sbjct 426 TATCGGGCCAGCATCAGTTTGGGCGGTAGGAGAATTGCGTTGGAATGTGGCACGGCTTCG 485

Query 481 GTTGTGTGTTATAGCCTTCGTCGATACTGCCAGCCTAGACTGAGGACTGCGGTTTATACC 540

||||||||||||||||||||||||||||||||||||||||||||||||||||||||||||

Sbjct 486 GTTGTGTGTTATAGCCTTCGTCGATACTGCCAGCCTAGACTGAGGACTGCGGTTTATACC 545

Query 541 TAGGATGTTGGCATAATGATCTTAAGTCGCCCGTCTTG 578

||||||||||||||||||||||||||||||||||||||

Sbjct 546 TAGGATGTTGGCATAATGATCTTAAGTCGCCCGTCTTG 583

**>MH545915 *Candida tropicalis* strain CBS 1920** 18S small subunit ribosomal RNA gene, partial sequence; internal transcribed spacer 1, 5.8S ribosomal RNA gene, and internal transcribed spacer 2, complete sequence; and *26S* large subunit ribosomal RNA gene, partial sequence

Sequence ID: MH545915.1Length: 2785Number of Matches: 1

Range 1: 2136 to 2713GenBankGraphicsNext MatchPrevious Match

Alignment statistics for match #1

Score Expect Identities Gaps Strand

1068 bits (578) 0.0 578/578 (100%) 0/578 (0%) Plus/Plus

**Query = Pu-T2, Pu-T4 and Sw-T1**

**Sbject = MH545915 *Candida tropicalis* strain CBS 1920**

Query 1 AAACCAACAGGGATTGCCTTAGTAGCGGCGAGTGAAGCGGCAAAAGCTCAAATTTGAAAT 60

||||||||||||||||||||||||||||||||||||||||||||||||||||||||||||

Sbjct 2136 AAACCAACAGGGATTGCCTTAGTAGCGGCGAGTGAAGCGGCAAAAGCTCAAATTTGAAAT 2195

Query 61 CTGGCTCTTTCAGAGTCCGAGTTGTAATTTGAAGAAGGTATCTTTGGGTCTGGCTCTTGT 120

||||||||||||||||||||||||||||||||||||||||||||||||||||||||||||

Sbjct 2196 CTGGCTCTTTCAGAGTCCGAGTTGTAATTTGAAGAAGGTATCTTTGGGTCTGGCTCTTGT 2255

Query 121 CTATGTTTCTTGGAACAGAACGTCACAGAGGGTGAGAATCCCGTGCGATGAGATGATCCA 180

||||||||||||||||||||||||||||||||||||||||||||||||||||||||||||

Sbjct 2256 CTATGTTTCTTGGAACAGAACGTCACAGAGGGTGAGAATCCCGTGCGATGAGATGATCCA 2315

Query 181 GGCCTATGTAAAGTTCCTTCGAAGAGTCGAGTTGTTTGGGAATGCAGCTCTAAGTGGGTG 240

||||||||||||||||||||||||||||||||||||||||||||||||||||||||||||

Sbjct 2316 GGCCTATGTAAAGTTCCTTCGAAGAGTCGAGTTGTTTGGGAATGCAGCTCTAAGTGGGTG 2375

Query 241 GTAAATTCCATCTAAAGCTAAATATTGGCGAGAGACCGATAGCGAACAAGTACAGTGATG 300

||||||||||||||||||||||||||||||||||||||||||||||||||||||||||||

Sbjct 2376 GTAAATTCCATCTAAAGCTAAATATTGGCGAGAGACCGATAGCGAACAAGTACAGTGATG 2435

Query 301 GAAAGATGAAAAGAACTTTGAAAAGAGAGTGAAAAAGTACGTGAAATTGTTGAAAGGGAA 360

||||||||||||||||||||||||||||||||||||||||||||||||||||||||||||

Sbjct 2436 GAAAGATGAAAAGAACTTTGAAAAGAGAGTGAAAAAGTACGTGAAATTGTTGAAAGGGAA 2495

Query 361 GGGCTTGAGATCAGACTTGGTATTTTGTATGTTACTTCTTCGGGGGTGGCCTCTACAGTT 420

||||||||||||||||||||||||||||||||||||||||||||||||||||||||||||

Sbjct 2496 GGGCTTGAGATCAGACTTGGTATTTTGTATGTTACTTCTTCGGGGGTGGCCTCTACAGTT 2555

Query 421 TATCGGGCCAGCATCAGTTTGGGCGGTAGGAGAATTGCGTTGGAATGTGGCACGGCTTCG 480

||||||||||||||||||||||||||||||||||||||||||||||||||||||||||||

Sbjct 2556 TATCGGGCCAGCATCAGTTTGGGCGGTAGGAGAATTGCGTTGGAATGTGGCACGGCTTCG 2615

Query 481 GTTGTGTGTTATAGCCTTCGTCGATACTGCCAGCCTAGACTGAGGACTGCGGTTTATACC 540

||||||||||||||||||||||||||||||||||||||||||||||||||||||||||||

Sbjct 2616 GTTGTGTGTTATAGCCTTCGTCGATACTGCCAGCCTAGACTGAGGACTGCGGTTTATACC 2675

Query 541 TAGGATGTTGGCATAATGATCTTAAGTCGCCCGTCTTG 578

||||||||||||||||||||||||||||||||||||||

Sbjct 2676 TAGGATGTTGGCATAATGATCTTAAGTCGCCCGTCTTG 2713

**>MN421010 *Candida tropicalis* strain LY_15** large subunit ribosomal RNA gene, partial sequence Sequence ID: MN421010.1Length: 589Number of Matches: 1

Range 1: 10 to 587GenBankGraphicsNext MatchPrevious Match

Alignment statistics for match #1

Score Expect Identities Gaps Strand

1068 bits (578) 0.0 578/578 (100%) 0/578 (0%) Plus/Plus

**Query = Pu-T2, Pu-T4 and Sw-T1**

**Sbject = MN421010 *Candida tropicalis* strain LY_15**

Query 1 AAACCAACAGGGATTGCCTTAGTAGCGGCGAGTGAAGCGGCAAAAGCTCAAATTTGAAAT 60

||||||||||||||||||||||||||||||||||||||||||||||||||||||||||||

Sbjct 10 AAACCAACAGGGATTGCCTTAGTAGCGGCGAGTGAAGCGGCAAAAGCTCAAATTTGAAAT 69

Query 61 CTGGCTCTTTCAGAGTCCGAGTTGTAATTTGAAGAAGGTATCTTTGGGTCTGGCTCTTGT 120

||||||||||||||||||||||||||||||||||||||||||||||||||||||||||||

Sbjct 70 CTGGCTCTTTCAGAGTCCGAGTTGTAATTTGAAGAAGGTATCTTTGGGTCTGGCTCTTGT 129

Query 121 CTATGTTTCTTGGAACAGAACGTCACAGAGGGTGAGAATCCCGTGCGATGAGATGATCCA 180

||||||||||||||||||||||||||||||||||||||||||||||||||||||||||||

Sbjct 130 CTATGTTTCTTGGAACAGAACGTCACAGAGGGTGAGAATCCCGTGCGATGAGATGATCCA 189

Query 181 GGCCTATGTAAAGTTCCTTCGAAGAGTCGAGTTGTTTGGGAATGCAGCTCTAAGTGGGTG 240

||||||||||||||||||||||||||||||||||||||||||||||||||||||||||||

Sbjct 190 GGCCTATGTAAAGTTCCTTCGAAGAGTCGAGTTGTTTGGGAATGCAGCTCTAAGTGGGTG 249

Query 241 GTAAATTCCATCTAAAGCTAAATATTGGCGAGAGACCGATAGCGAACAAGTACAGTGATG 300

||||||||||||||||||||||||||||||||||||||||||||||||||||||||||||

Sbjct 250 GTAAATTCCATCTAAAGCTAAATATTGGCGAGAGACCGATAGCGAACAAGTACAGTGATG 309

Query 301 GAAAGATGAAAAGAACTTTGAAAAGAGAGTGAAAAAGTACGTGAAATTGTTGAAAGGGAA 360

||||||||||||||||||||||||||||||||||||||||||||||||||||||||||||

Sbjct 310 GAAAGATGAAAAGAACTTTGAAAAGAGAGTGAAAAAGTACGTGAAATTGTTGAAAGGGAA 369

Query 361 GGGCTTGAGATCAGACTTGGTATTTTGTATGTTACTTCTTCGGGGGTGGCCTCTACAGTT 420

||||||||||||||||||||||||||||||||||||||||||||||||||||||||||||

Sbjct 370 GGGCTTGAGATCAGACTTGGTATTTTGTATGTTACTTCTTCGGGGGTGGCCTCTACAGTT 429

Query 421 TATCGGGCCAGCATCAGTTTGGGCGGTAGGAGAATTGCGTTGGAATGTGGCACGGCTTCG 480

||||||||||||||||||||||||||||||||||||||||||||||||||||||||||||

Sbjct 430 TATCGGGCCAGCATCAGTTTGGGCGGTAGGAGAATTGCGTTGGAATGTGGCACGGCTTCG 489

Query 481 GTTGTGTGTTATAGCCTTCGTCGATACTGCCAGCCTAGACTGAGGACTGCGGTTTATACC 540

||||||||||||||||||||||||||||||||||||||||||||||||||||||||||||

Sbjct 490 GTTGTGTGTTATAGCCTTCGTCGATACTGCCAGCCTAGACTGAGGACTGCGGTTTATACC 549

Query 541 TAGGATGTTGGCATAATGATCTTAAGTCGCCCGTCTTG 578

||||||||||||||||||||||||||||||||||||||

Sbjct 550 TAGGATGTTGGCATAATGATCTTAAGTCGCCCGTCTTG 587

**>MN421006 *Candida tropicalis* strain LY_11** large subunit ribosomal RNA gene, partial sequence

Sequence ID: MN421006.1Length: 592Number of Matches: 1

Range 1: 13 to 590GenBankGraphicsNext MatchPrevious Match

Alignment statistics for match #1

Score Expect Identities Gaps Strand

1068 bits (578) 0.0 578/578 (100%) 0/578 (0%) Plus/Plus

**Query = Pu-T2, Pu-T4 and Sw-T1**

**Sbject = MN421006 *Candida tropicalis* strain LY_11**

Query 1 AAACCAACAGGGATTGCCTTAGTAGCGGCGAGTGAAGCGGCAAAAGCTCAAATTTGAAAT 60

||||||||||||||||||||||||||||||||||||||||||||||||||||||||||||

Sbjct 13 AAACCAACAGGGATTGCCTTAGTAGCGGCGAGTGAAGCGGCAAAAGCTCAAATTTGAAAT 72

Query 61 CTGGCTCTTTCAGAGTCCGAGTTGTAATTTGAAGAAGGTATCTTTGGGTCTGGCTCTTGT 120

||||||||||||||||||||||||||||||||||||||||||||||||||||||||||||

Sbjct 73 CTGGCTCTTTCAGAGTCCGAGTTGTAATTTGAAGAAGGTATCTTTGGGTCTGGCTCTTGT 132

Query 121 CTATGTTTCTTGGAACAGAACGTCACAGAGGGTGAGAATCCCGTGCGATGAGATGATCCA 180

||||||||||||||||||||||||||||||||||||||||||||||||||||||||||||

Sbjct 133 CTATGTTTCTTGGAACAGAACGTCACAGAGGGTGAGAATCCCGTGCGATGAGATGATCCA 192

Query 181 GGCCTATGTAAAGTTCCTTCGAAGAGTCGAGTTGTTTGGGAATGCAGCTCTAAGTGGGTG 240

||||||||||||||||||||||||||||||||||||||||||||||||||||||||||||

Sbjct 193 GGCCTATGTAAAGTTCCTTCGAAGAGTCGAGTTGTTTGGGAATGCAGCTCTAAGTGGGTG 252

Query 241 GTAAATTCCATCTAAAGCTAAATATTGGCGAGAGACCGATAGCGAACAAGTACAGTGATG 300

||||||||||||||||||||||||||||||||||||||||||||||||||||||||||||

Sbjct 253 GTAAATTCCATCTAAAGCTAAATATTGGCGAGAGACCGATAGCGAACAAGTACAGTGATG 312

Query 301 GAAAGATGAAAAGAACTTTGAAAAGAGAGTGAAAAAGTACGTGAAATTGTTGAAAGGGAA 360

||||||||||||||||||||||||||||||||||||||||||||||||||||||||||||

Sbjct 313 GAAAGATGAAAAGAACTTTGAAAAGAGAGTGAAAAAGTACGTGAAATTGTTGAAAGGGAA 372

Query 361 GGGCTTGAGATCAGACTTGGTATTTTGTATGTTACTTCTTCGGGGGTGGCCTCTACAGTT 420

||||||||||||||||||||||||||||||||||||||||||||||||||||||||||||

Sbjct 373 GGGCTTGAGATCAGACTTGGTATTTTGTATGTTACTTCTTCGGGGGTGGCCTCTACAGTT 432

Query 421 TATCGGGCCAGCATCAGTTTGGGCGGTAGGAGAATTGCGTTGGAATGTGGCACGGCTTCG 480

||||||||||||||||||||||||||||||||||||||||||||||||||||||||||||

Sbjct 433 TATCGGGCCAGCATCAGTTTGGGCGGTAGGAGAATTGCGTTGGAATGTGGCACGGCTTCG 492

Query 481 GTTGTGTGTTATAGCCTTCGTCGATACTGCCAGCCTAGACTGAGGACTGCGGTTTATACC 540

||||||||||||||||||||||||||||||||||||||||||||||||||||||||||||

Sbjct 493 GTTGTGTGTTATAGCCTTCGTCGATACTGCCAGCCTAGACTGAGGACTGCGGTTTATACC 552

Query 541 TAGGATGTTGGCATAATGATCTTAAGTCGCCCGTCTTG 578

||||||||||||||||||||||||||||||||||||||

Sbjct 553 TAGGATGTTGGCATAATGATCTTAAGTCGCCCGTCTTG 590

**>MN421005 *Candida tropicalis* strain LY_7** large subunit ribosomal RNA gene, partial sequence

Sequence ID: MN421005.1Length: 589Number of Matches: 1

Range 1: 10 to 587GenBankGraphicsNext MatchPrevious Match

Alignment statistics for match #1

Score Expect Identities Gaps Strand

1068 bits (578) 0.0 578/578 (100%) 0/578 (0%) Plus/Plus

**Query = Pu-T2, Pu-T4 and Sw-T1**

**Sbject = MN421005 *Candida tropicalis* strain LY_7**

Query 1 AAACCAACAGGGATTGCCTTAGTAGCGGCGAGTGAAGCGGCAAAAGCTCAAATTTGAAAT 60

||||||||||||||||||||||||||||||||||||||||||||||||||||||||||||

Sbjct 10 AAACCAACAGGGATTGCCTTAGTAGCGGCGAGTGAAGCGGCAAAAGCTCAAATTTGAAAT 69

Query 61 CTGGCTCTTTCAGAGTCCGAGTTGTAATTTGAAGAAGGTATCTTTGGGTCTGGCTCTTGT 120

||||||||||||||||||||||||||||||||||||||||||||||||||||||||||||

Sbjct 70 CTGGCTCTTTCAGAGTCCGAGTTGTAATTTGAAGAAGGTATCTTTGGGTCTGGCTCTTGT 129

Query 121 CTATGTTTCTTGGAACAGAACGTCACAGAGGGTGAGAATCCCGTGCGATGAGATGATCCA 180

||||||||||||||||||||||||||||||||||||||||||||||||||||||||||||

Sbjct 130 CTATGTTTCTTGGAACAGAACGTCACAGAGGGTGAGAATCCCGTGCGATGAGATGATCCA 189

Query 181 GGCCTATGTAAAGTTCCTTCGAAGAGTCGAGTTGTTTGGGAATGCAGCTCTAAGTGGGTG 240

||||||||||||||||||||||||||||||||||||||||||||||||||||||||||||

Sbjct 190 GGCCTATGTAAAGTTCCTTCGAAGAGTCGAGTTGTTTGGGAATGCAGCTCTAAGTGGGTG 249

Query 241 GTAAATTCCATCTAAAGCTAAATATTGGCGAGAGACCGATAGCGAACAAGTACAGTGATG 300

||||||||||||||||||||||||||||||||||||||||||||||||||||||||||||

Sbjct 250 GTAAATTCCATCTAAAGCTAAATATTGGCGAGAGACCGATAGCGAACAAGTACAGTGATG 309

Query 301 GAAAGATGAAAAGAACTTTGAAAAGAGAGTGAAAAAGTACGTGAAATTGTTGAAAGGGAA 360

||||||||||||||||||||||||||||||||||||||||||||||||||||||||||||

Sbjct 310 GAAAGATGAAAAGAACTTTGAAAAGAGAGTGAAAAAGTACGTGAAATTGTTGAAAGGGAA 369

Query 361 GGGCTTGAGATCAGACTTGGTATTTTGTATGTTACTTCTTCGGGGGTGGCCTCTACAGTT 420

||||||||||||||||||||||||||||||||||||||||||||||||||||||||||||

Sbjct 370 GGGCTTGAGATCAGACTTGGTATTTTGTATGTTACTTCTTCGGGGGTGGCCTCTACAGTT 429

Query 421 TATCGGGCCAGCATCAGTTTGGGCGGTAGGAGAATTGCGTTGGAATGTGGCACGGCTTCG 480

||||||||||||||||||||||||||||||||||||||||||||||||||||||||||||

Sbjct 430 TATCGGGCCAGCATCAGTTTGGGCGGTAGGAGAATTGCGTTGGAATGTGGCACGGCTTCG 489

Query 481 GTTGTGTGTTATAGCCTTCGTCGATACTGCCAGCCTAGACTGAGGACTGCGGTTTATACC 540

||||||||||||||||||||||||||||||||||||||||||||||||||||||||||||

Sbjct 490 GTTGTGTGTTATAGCCTTCGTCGATACTGCCAGCCTAGACTGAGGACTGCGGTTTATACC 549

Query 541 TAGGATGTTGGCATAATGATCTTAAGTCGCCCGTCTTG 578

||||||||||||||||||||||||||||||||||||||

Sbjct 550 TAGGATGTTGGCATAATGATCTTAAGTCGCCCGTCTTG 587
