# Supplementary material for: Changes in the bulk soil after fresh corn grown with organic and inorganic fertilizer application
Source: PLoS One. 2025 Jul 10;20(7):e0326730. doi: 10.1371/journal.pone.0326730 (PMC12244754; doi:10.1371/journal.pone.0326730)
Supplement: S2 File — (DOC) [file pone.0326730.s002.doc]

**S3 Link of PSF sequences deposited in NCBI**

| **Accession number** | **Link** |
| --- | --- |
| SUB14477274 Sw-T1 PP848218 | <https://submit.ncbi.nlm.nih.gov/subs/?search=SUB14477274> |
| SUB14459899 Pu-T2 PP814954 | <https://submit.ncbi.nlm.nih.gov/subs/?search=SUB14459899> |
| SUB14477270 Pu-T4 PP848217 | <https://submit.ncbi.nlm.nih.gov/subs/?search=SUB14477270> |
